# Lysosomal pH Is Regulated in a Sex Dependent Manner in Immune Cells Expressing *CXorf21*

**DOI:** 10.3389/fimmu.2019.00578

**Published:** 2019-04-02

**Authors:** Valerie M. Harris, Isaac T. W. Harley, Biji T. Kurien, Kristi A. Koelsch, Robert Hal Scofield

**Affiliations:** ^1^Arthritis and Clinical Immunology Program, Oklahoma Medical Research Foundation, Oklahoma City, OK, United States; ^2^Department of Pathology, College of Medicine, University of Oklahoma Health Sciences Center, Oklahoma City, OK, United States; ^3^Department of Medicine, College of Medicine, University of Oklahoma Health Sciences Center, Oklahoma City, OK, United States; ^4^Research Service, Oklahoma City Department of Veterans Affairs Health Care Center, Oklahoma City, OK, United States; ^5^Division of Rheumatology, University of Colorado School of Medicine, Aurora, CO, United States; ^6^Department of Immunology & Microbiology, University of Colorado School of Medicine, Aurora, CO, United States; ^7^Medical Service, Oklahoma City Department of Veterans Affairs Health Care Center, Oklahoma City, OK, United States

**Keywords:** systemic lupus erythematosus, Sjögren's syndrome, lysosome, pH, X chromosome, sex bias

## Abstract

**Background:** *CXorf21* and *SLC15a4* both contain risk alleles for systemic lupus erythematosus (SLE) and Sjögren's syndrome (pSS). The former escapes X inactivation. Our group predicts specific endolysosomal-dependent immune responses are driven by the protein products of these genes, which form a complex at the endolysosomal surface. Our previous studies have shown that knocking out *CXorf21* increases lysosomal pH in female monocytes, and the present study assesses whether the lysosomal pH in 46,XX women, who overexpress CXorf21 in monocytes, B cells, and dendritic cells (DCs), differs from 46,XY men.

**Methods:** To determine endolysosome compartment pH we used both LysoSensor™ Yellow/Blue DND-160 and pHrodo® Red AM Intracellular pH Indicator in primary monocyte, B cells, DCs, NK cells, and T cells from healthy men and women volunteers.

**Results:** Compared to male samples, female monocytes, B cells, and DCs had lower endolysosomal pH (female/male pH value: monocytes 4.9/5.6 *p* < 0.0001; DCs 4.9/5.7 *p* = 0.044; B cells 5.0/5.6 *p* < 0.05). Interestingly, T cells and NK cells, which both express low levels of CXorf21, showed no differential pH levels between men and women.

**Conclusion:** We have previously shown that subjects with two or more X-chromosomes have increased CXorf21 expression in specific primary immune cells. Moreover, knockdown of CXorf21 increases lysosomal pH in female monocytes. The present data show that female monocytes, DC, B cells, where CXorf21 is robustly expressed, have lower lysosomal pH compared to the same immune cell populations from males. The lower pH levels observed in specific female immune cells provide a function to these SLE/SS-associated genes and a mechanism for the reported inflated endolysosomal-dependent immune response observed in women compared to men (i.e., TLR7/type I Interferon activity).

## Introduction

Systemic lupus erythematosus (SLE) and Sjögren's syndrome (SS) are chronic autoimmune diseases that are highly related in both clinical and serological manifestations. In terms of the latter, autoantibodies binding the Ro/La (or SSA/SSB) ribonucleoprotein particle are found in about half of patients with SLE and up to 80% of those with SS (1). Like most autoimmune diseases, both SLE and SS much more commonly affect women than men with ratios of about 10 to 1 for SLE, and up to 15 to 1 women to men in SS (2). While either disease can have its onset throughout the entire lifespan, the peak age of onset for SLE is about 30, while that for SS is in older adulthood.

The diseases are also related in regards to pathophysiology. For instance, most risk genes identified in genome wide association studies are shared between SLE and SS (3–6). Pertinent to the work presented herein, another aspect of shared pathophysiology involves interferon. There is increased expression of interferon-regulated genes in peripheral blood mononuclear cells from patients with either disease (7–10). Evidence from both human disease (11–13) and murine models (14–17) suggests that signaling through lysosomal, nucleic acid-binding toll-like receptors (TLR) 7 and 9 is in part responsible for the pathogenicity, including increased interferon activity in these diseases.

Signaling through stimulation of intracellular TLRs is exquisitely sensitive to lysosomal pH. The soluble carrier protein 15a4 (*SLC15a4*) and *CXorf21* genes have been identified as containing risk alleles for both SLE and SS (3, 6, 18). The protein products of these genes are binding partners on the lysosomal membrane (19). The SLC15a4 protein participates in movement of hydrogen ion and oligopeptides in and out of the lysosome. Thus, SLC15a4 regulates antigen processing in the lysosome along with TLR7/9–mediated cytokine secretion, NF-κB signaling and antibody production (20, 21). The regulatory role of SLC15a4 is at least in part mediated by control of lysosomal pH (21). A loss of function *Slc15a4* mutation ameliorates murine lupus and impairs interferon production mediated through TLR7 stimulation (20). An allele within *CXorf21* was recently identified as a lupus risk gene (18). Our data demonstrate the CXorf21 protein is expressed in only monocytes, B lymphocytes and dendritic cells. In addition, CXorf21 routinely escapes X inactivation (22) with both mRNA and protein levels higher in female cells compared to male cells (Harris et al., unpublished). CXorf21 knockdown using small guide RNA resulted in an abrogation of interferon production after exposure of female cells to TLR7 agonist. In addition, we found an increased lysosomal pH in female cells with CXorf21 knockdown (Harris et al., unpublished).

While there have been many theories concerning the increased risk for autoimmune disease in women, based on studies of X chromosome aneuploidies in subjects with SLE or SS, we propose that the female risk of SLE and SS is a result of a dose effect for the X chromosome. Our previous data show that Klinefelter men (47,XXY) are enriched 30-fold among men with either SLE or SS (23, 24). Also, SLE or SS affected women have an increased prevalence of 47,XXX compared to healthy control women or women with either rheumatoid arthritis or primary biliary cirrhosis (25). Because *CXorf21* escapes X inactivation; and, therefore, female cells have approximately twice the amount of CXorf21 protein, this gene is a candidate to mediate the X chromosome dose effect found for both SLE and SS, but not other studied, female-biased autoimmune diseases where no X dose effect was found.

We undertook the present study to further characterize the cellular function of CXorf21. In particular, the complex of SLC15a4 and CXorf21 affects lysosomal pH, and CXorf21 expression is greater in female cells compared to male cells. Thus, we sought to determine whether there is a difference in lysosomal pH between male and female immune cells, in which *CXorf21* is known to be expressed.

## Methods

### Patients/Donors

Whole blood was donated by volunteer healthy controls. Healthy female and male controls were recruited pair-wise to control for day-to-day variability. EBV-transformed B cells or lymphoblastoid cell lines (LCLs) derived from healthy controls or SLE patients with and without chromosomal aneuploidies were obtained from the Lupus Family Registry and Repository (26). Eight male and 8 female buffy coats were obtained from the Oklahoma Blood Institute (OBI) (Oklahoma City, OK). All donors were Caucasian with ages ranging between 28 and 45 years old. Healthy subjects had no known chronic medical illness and tested negative for OBI blood safety screening panel. Buffy coats were stored at room temperature until cell isolation. All subjects gave written informed consent in accordance with the Declaration of Helsinki. The protocol was approved by the University of Oklahoma Health Sciences Center Institutional Review Board.

### Isolation of Cells

STEMCELL EasySep™ monocyte, dendritic cells, B cell, natural killer cells (NK cells), and T cells were used to isolate monocytes, dendritic cells, B cells, NK cells, and T cells, respectively, from PBMCs of healthy controls. Briefly, PBMCs were first purified from buffy coats using density gradient centrifugation using Lymphoprep (STEMCELL Technologies, Cambridge, MA) according to the manufacturer's protocol. Cells were resuspended in EasySep™ buffer, the EasySep™ Magnet was used to sequentially isolate CD14^+^ (using the EasySep™ Human CD14 enrichment kit), CD19^+^ (using the EasySep Human CD19 positive selection kit II), CD3-CD56+ (EasySep™ Human NK Cell Isolation Kit) and CD3+ (EasySep™ Human T Cell Isolation Kit). Cell population purity was confirmed by Moxi-Flow cytometry with the protocol as described (27).

### Western Blot Analysis

SDS-PAGE was carried out according to Laemmli et al., except for using pre-cast 4–20% gradient gels (Bio-Rad). Gel proteins were transferred to nitrocellulose membranes using Trans-Blot Turbo transfer system and Trans-Blot Turbo transfer pack (Bio-Rad). Proteins were probed with anti-CXorf21 and anti-actin antibodies (Novus Biotechnologies) and detected with alkaline phosphatase/nitro blue tetrazolium/5-bromo-5-chloro-3-indolyl phosphate system. Protein bands were quantified using densitometry (ImageJ).

### Lysosomal pH Determination

To detect differences in intracellular pH in live human male and female primary monocytes, dendritic cells, B cells, NK cells, and T cells, the LysoSensor™ Yellow/Blue DND-160 (Thermofisher) and pHrodo® Red AM Intracellular pH Indicator (Thermofisher) was used according to the manufacturer's suggested protocol. Briefly, primary cells were treated with LysoSensor reagents as ratiometric means for measuring lysosomal pH via fluorescence. The ratio of fluorescence allows for the adjustment for possible variability between particle uptake. To quantitate pH, primary cells plated on a 96-well plate were loaded with 5 μM pHrodo® Red AM intracellular pH indicators for 30 min. Cells were then washed with a series of Live Cell Imaging Media™ and standard buffers containing 10 μM nigericin and 10 μM valinomycin were added for 5 min in order to clamp intracellular pH values 4.5, 5.5, 6.5, and 7.5. We determined the mean cellular fluorescence in triplicate samples using a spectrophotometer (Synergy H1, Biotek). A standard curve for male or female samples showed a linear relationship between the intracellular pH and the relative fluorescence units.

### Statistics

Statistical analyses were carried out using *T*-test, one-way ANOVA with multiple comparisons, or Fisher's Exact test using GraphPad Prism 7.

## Results

### Primary Female Human Monocytes Have a More Acidic Lysosomal pH Compared to Male Cells

**Figure 1:**
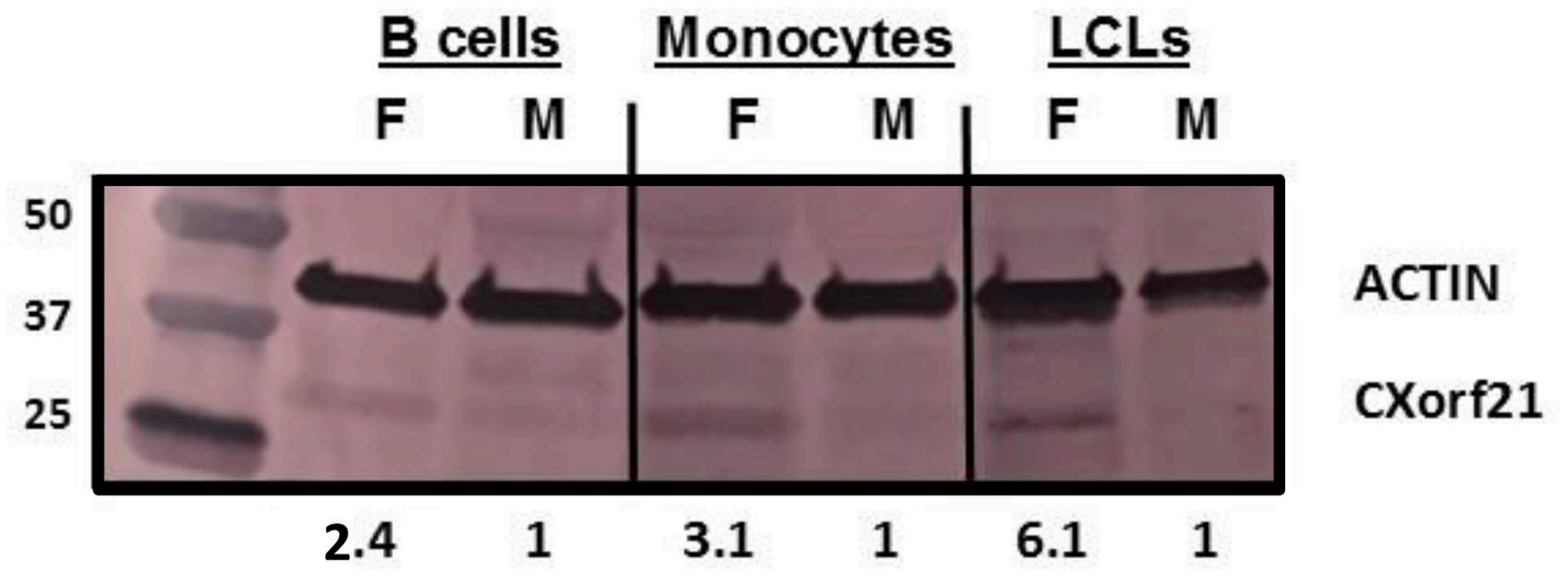
CXorf21 is differentially expressed in primary immune cells of healthy controls and SLE-affected patients. Total protein extract was harvested from healthy male and female primary B cells (lane 1 and 2), primary monocytes (lanes 3 and 4), and lymphoblastoid cell lines (LCLs) (lane 5 and 6) and subjected to SDS-PAGE. Western blotting using human anti-CXorf21 antibody (34 kD) identifying bands at the appropriate molecular weight. Human anti-actin (42 kD) is shown as a loading control. Densitometry via Image J was used to quantitative optical density of protein bands.

**Figure 2:**
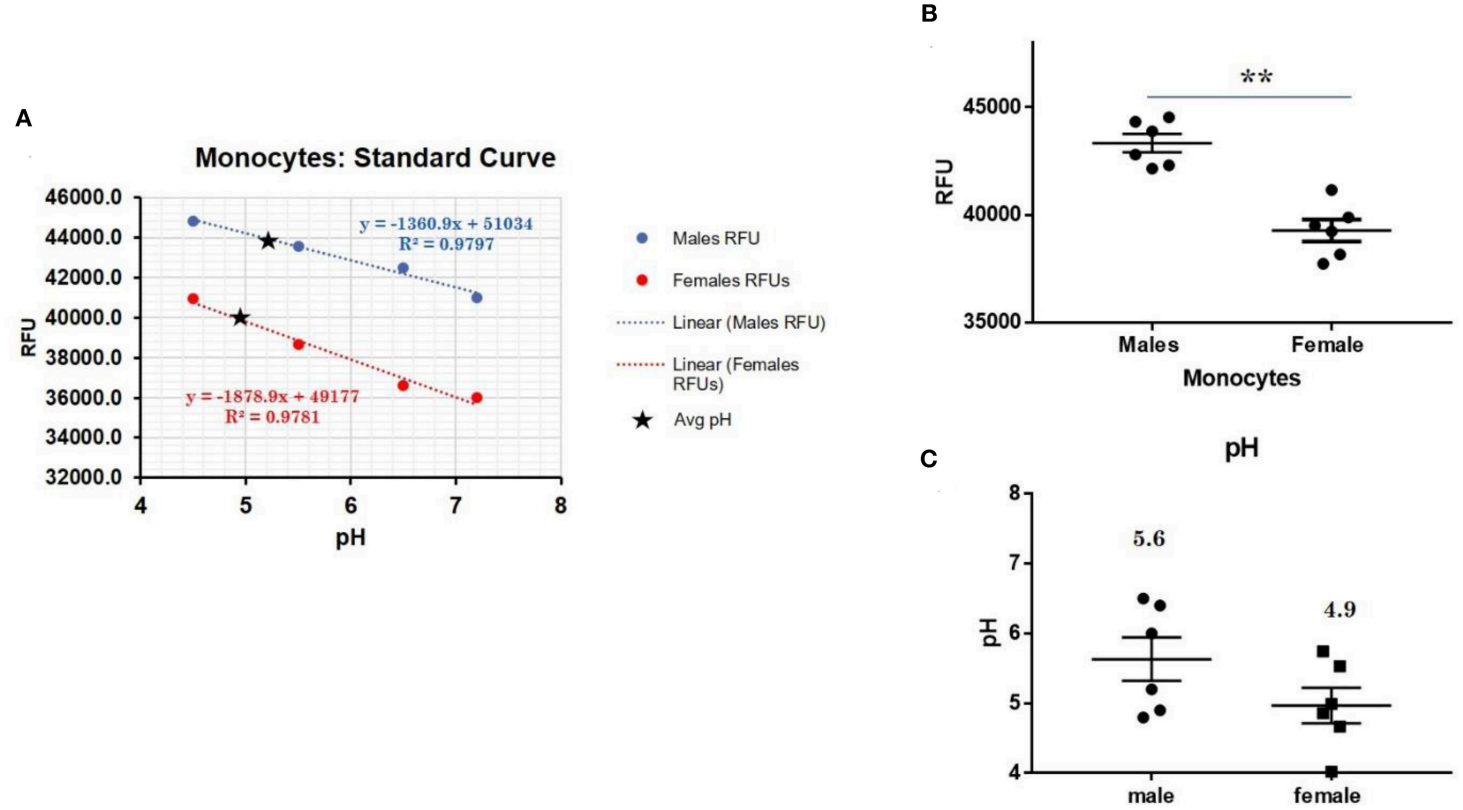
Differences in lysosomal pH in female and male monocytes. **(A)** Male (*n* = 6) and female monocytes (*n* = 6) standard curve using pHrodo^TM^ Red AM with Intracellular pH Calibration Buffer Kit for the translation of fluorescence ratios into pH. An average of six data points was plotted in the graph and a linear trend line was fitted to get a pH standard curve. **(B)** Male monocytes female monocytes were stained with pHrodo^TM^ Red AM solution and relative fluorescence units (RFUs) were measured with multi-well plate reader (Details in Materials and Methods). **(C)** pH determined using pHrodo indication kit according to manufactures protocol. Student's *t*-test was used to determine statistical difference in RFUs that was converted into pH values via each standard curve. ^**^p = 0.0001; Error bar represent SEM.

CXorf21 mRNA and protein are expressed at higher levels in primary monocytes, CD19+ B cells, and Lymphoblastoid cell lines (LCLs) from female healthy subjects compared to male controls (Figure 1: Harris et al., unpublished). Based on the function of the CXorf21 protein and its interaction with SLC15A4, which is known to regulate lysosomal pH, we hypothesized that, with greater expression of the CXorf21 protein, lysosomal pH would be lower in female male monocytes. In order to assess a difference in lysosomal pH between male and female monocytes, we performed *ex vivo* lysosomal pH measurements. Following a 30-min incubation period of the cells with pH calibration buffers, a standard curve for both male and female (Figure 2A) primary monocytes to determine pH based on male and female (Figure 2B) relative fluorescence was generated. We found that unstimulated female monocytes have a significantly more acidic lysosomal pH (4.9) compared to male monocytes (5.6) (Figure 2C) (*p* = 0.0001 by Fisher's exact test).

### Other Cell Types in Which CXorf21 Is Expressed Higher in Female Cells

**Figure 3:**
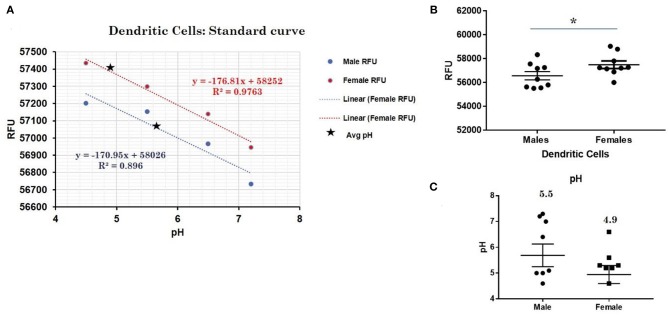
Differences in lysosomal pH in female and male dendritic cells. **(A)** Male (*n* = 6) and female dendritic cells (*n* = 6) standard curve using pHrodo^TM^ Red AM with Intracellular pH Calibration Buffer Kit for the translation of fluorescence ratios into pH. An average of six data points was plotted in the graph and a linear trend line was fitted to get a pH standard curve. **(B)** Male DCs or female DCs were stained with pHrodo^TM^ Red AM solution and relative fluorescence units (RFUs) were measured with multi-well plate reader (Details in Material and Methods). **(C)** pH determined using pHrodo indication kit according to manufactures protocol. Student's *t*-test was used to determine statistical difference in RFUs that was converted into pH values via each standard curve. ^*^p-value 0.044; Error bar represent SEM.

**Figure 4:**
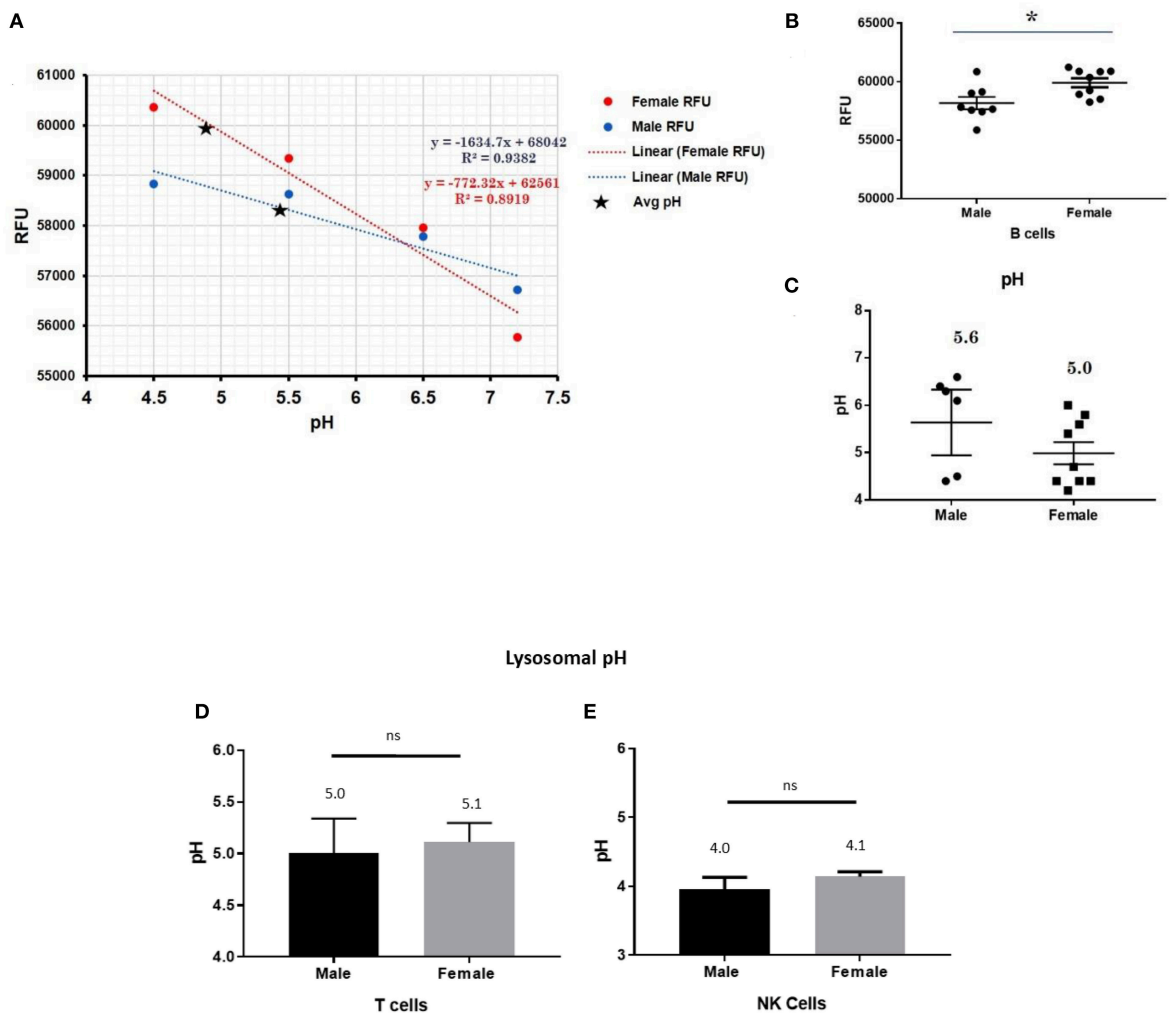
Differences in lysosomal pH in female and male CD19+ B cells, pan-T cells, and NK cells. **(A)** Male and female B cells (*n* = 6) standard curve using pHrodo^TM^ Red AM with Intracellular pH Calibration Buffer Kit for the translation of fluorescence ratios into pH. An average of six data points was plotted in the graph and a linear trend line was fitted to get a pH standard curve. **(B)** Male B cells or female B cells were stained with pHrodo^TM^ Red AM solution and relative fluorescence units (RFUs) were measured with multi-well plate reader (Details in Material and Methods). **(C)** pH determined using pHrodo indication kit according to manufactures protocol. Male and Female **(D)** pan-T cells and **(E)** NK cell pH was determined as described above (standard curves and RFUs data not shown) Student's *t*-test was used to determine statistical difference in RFUs and pH that was converted into pH values via each standard curve; ^*^p < 0.05; ns=not significant; Error bar represent SEM.

This trend for lower pH in the lysosomes of female cells held true for both dendritic cells, where female DCs lysosomal pH was 4.9 and male DCs were 5.7 (*p-value* 0.044; Figure 3), as well as B lymphocytes, where female lysosomal pH was 5.0 and male lysosomal pH was 5.6 (*p* = 0.0447 by Fisher's exact test; Figure 4). These data suggest that female monocytes, DCs, and B cells, immune cells with increased CXorf21 and TLR7 expression, have a more favorable lysosomal processing environment compared to male cells, and may drive the robust TLRs/lysosomal-dependent immune response observed women compared to men (28).

### Lysosomal pH in Cells That do Not Express CXorf21

In order to assess the role of female overexpression of *CXorf21* in lysosomal pH, we studied immune cells in which the expression of *CXorf21* is absent. To that end, we isolated primary T cell and NK cells, two immune cells with minimal CXorf21 and TLR7 levels. We found, while their lysosomal pH was optimal for lysosomal signaling, there was no significant difference in pH between the sexes (Figures 4D,E).

## Discussion

There are ~80 autoimmune disease, the great majority of which affect women more than men. Both SLE and SS have a ratio of about 10 affected women for every one affected man (2). The sex bias in SLE is present among patients with childhood onset (29). In SLE and SS, more women are affected than men in older adulthood at ages where women are post-menopausal (30). Despite much investigation, a compelling explanation for this sex bias has not been forthcoming. Skewing of X chromosome inactivation, acquired X chromosome monosomy, sex hormone levels have all been studied and found to not explain the sex bias (30–34). SS is much less well studied than SLE; however, again no explanation of the marked predilection for women has been made.

Based on data concerning X chromosome aneuploidies, we have proposed that the increased female risk of SLE and SS is the result of the X chromosome complement. Men with Klinefelter's syndrome (47,XXY) are enriched 15-fold in these diseases (23, 24). In addition, 47,XXX women are also found in excess among those with either SLE or SS, but not rheumatoid arthritis or primary biliary cirrhosis (25). Very rare abnormalities of the X chromosome among patients with SLE or SS, including partial triplications, as well as rare patients with Turner's syndrome and SLE may localize the effect to the X chromosome distal p arm (35, 36).

Of course, in cells with two or more X chromosomes all but one X is inactivated in order to equalize gene dose compared to male cells. However, X inactivation is not an all or none phenomenon with a significant fraction (up to 20%) of X-linked human genes escaping X inactivation (22, 37). Thus, a key factor in the idea that an X chromosome gene dose effect mediates the sex bias of SLE and SS is the escape of X inactivation such that female cells have bi-allelic mRNA expression and potentially more functional protein of a given X-linked gene.

Two genes that escape X inactivation in immune cells, contain SLE-risk alleles, and have critical roles in production of interferon are *CXorf21* and *TLR7* (38). Thus, on this basis, these genes are candidates to mediate the X chromosome gene dose effect for the sex bias of SLE and SS. CXorf21 is a binding partner of another SLE-risk gene—*Slc15a4*. As noted above SLC15A4 is involved in transport of oligopeptides and hydrogen ions out of the lysosome, and knockout of *Slc15a4* results in abrogation of TLR7 signaling as well as amelioration of murine lupus (17, 18, 20, 21). We have shown that CXorf21 protein is expressed exclusively in monocytes, B cells, and dendritic cells, and the protein levels are two–three-fold higher in female cells compared to male cells. In addition knockdown of CXorf21 with CRISPR-Cas resulted in abrogation of interferon, TNF-α and IL6 secretion after TLR7 activation in female cells. Furthermore, lysosomal pH increased, suggesting an environment less conducive to lysosomal signaling (Harris et al., unpublished).

Because lysosomal pH was affected by decreased expression of CXorf21 and because female cells express more CXorf21 than do male cells, we hypothesized that female cells expressing CXorf21 would have a more acidic pH than male cells. Therefore, we studied lysosomal pH in B cells, monocytes and dendritic cells from healthy human subjects. In fact, the present results demonstrate that female cells had a more acidic lysosomal pH than did these cells from male subjects. T lymphocytes and NK cells, which do not express CXorf21 at appreciable levels, did not have a pH difference between male and female derived cells. Thus, we conclude that a possible functional role of CXorf21 is regulation of lysosomal pH, and that differing levels of expression between the sexes lead to distinct lysosomal pH. To our knowledge this is the first report of a lysosomal pH difference between male and female immune cells.

We have ventured to predict a mechanism of action for the uncharacterized protein CXorf21 as a short-chain dehydrogenase reductase. We put forth two plausible functions: (1) as a reductase CXorf21 utilizes NADPH to generate hydrogen ions for lysosome proton pump consumption (i.e., v-ATPase pump); or, (2) as a dehydrogenase generates NAPDH for lysosome superoxide production by the lysosomal-resident NOX2 complex. Both scenarios could result in changes in lysosomal pH.

What might be the functional consequences of this pH difference? Simply, altered (auto)antigen processing and presentation or modulation of endolysosomal resident TLR7 activity. As noted above, a recent report demonstrates that the X-linked *TLR7* gene is bi-allelically expressed in immune cells (that is, escapes X inactivation), and has increased protein levels in female cells compared to male cells (38). In addition to an increase in lysosomal pH and abrogation of TLR7 signaling, our data using *CXorf21* CRISPR-Cas knockdown show that there is a loss of TLR7 agonist-induced increased TLR7 expression (both mRNA and protein). Thus, CXorf21 is critically involved in TLR7 signaling, including a feedforward expression loop for TLR7. Thus, we propose increased expression of CXorf21, either because of the presence of two X chromosomes and the escape of X inactivation, or because the SLE-risk allele increases expression (18), leads to increased TLR7 signaling and increased interferon production.

The present study is limited, especially in regard to studying lysosomal pH and its regulation in regards to the pathogenesis and treatment of SLE. Endosomal TLR signaling, which leads to type 1 interferon production, is clearly important in SLE pathogenesis, both in humans and animal models (39). Furthermore, this signaling is exquisitely sensitive to changes in endolysosomal pH. *CXorf21, Slc15a4, TLR7*, and *NCF1* (encodes the p47phox NOX2 subunit) all contain SLE risk alleles (4–6). Published data discussed above demonstrate that SLC15A4 regulates endolysosomal pH, and data herein show that the protein product of *CXorf21* also regulates this pH. NOX2 is activated by phosphorylation of its p47phox subunit by TLR7 signaling (40, 41). Meanwhile, TLR7 and NOX2 signaling are both regulated by endolysosomal pH, and activated NOX2 regulates endolysosomal pH (42, 43). Both *CXorf21* and *TLR7* escape X inactivation (22). Thus, determining how these genes, all of which are involved in endososomal TLR signaling and type 1 interferon production, impact the pathogenesis and sex bias of SLE will need a great deal more investigation. The treatment of SLE may also be impacted by the interaction of these genes, their SLE-associated alleles and their protein products. Hydroxychloroquine, an important mainstay of SLE therapy (44–49), has a variety of effects (50–54), including altering endolysosomal pH, antigen presentation, TLR signaling and cytokine production. Obviously, these mechanisms of action intersect with the TLR7 signaling pathway and the genes discussed above. We have not addressed how the SLE-associated alleles in these four genes might influence the efficacy of hydroxychloroquine, which will likely require study of healthy controls as well as SLE patients with various combinations of these alleles.

These data demonstrate the function of SLC15A4 and CXorf21, which form a complex that regulates lysosomal pH, and in turn regulates TLR7 signaling. Furthermore, based on the differential expression of *CXorf21* between the sexes, we have shown that lysosomal pH, a key factor in signaling in this cellular compartment, is different between men and women. This difference and the resulting functional immune differences may contribute to the X chromosome gene dose that underlies the sex bias of SLE and SS.

## Data Availability

All datasets generated for this study are included in the manuscript and/or the supplementary files.

## Author Contributions

VH designed the experiments, carried out the experiments, wrote the first draft, edited the paper, and approved the final version. RS designed the experiments, wrote the manuscript, and approved the final version. IH designed the experiments, edited the paper, and approved the final version. BK carried out experiments, edited the paper, and approved the final version. KK designed the experiments, edited the paper, and approved the final version.

### Conflict of Interest Statement

The authors declare that the research was conducted in the absence of any commercial or financial relationships that could be construed as a potential conflict of interest.
